# *Troponin T *isoform expression is modulated during Atlantic Halibut metamorphosis

**DOI:** 10.1186/1471-213X-7-71

**Published:** 2007-06-18

**Authors:** Marco A Campinho, Nádia Silva, Mari A Nowell, Lynda Llewellyn, Glen E Sweeney, Deborah M Power

**Affiliations:** 1 CCMAR, FERN, Universidade do Algarve, Campus de Gambelas, 8005-139 Faro, Portugal; 2 School of Biosciences, University of Wales, Museum Avenue CF11 3US Cardiff, UK

## Abstract

**Background:**

Flatfish metamorphosis is a thyroid hormone (TH) driven process which leads to a dramatic change from a symmetrical larva to an asymmetrical juvenile. The effect of THs on muscle and in particular muscle sarcomer protein genes is largely unexplored in fish. The change in *Troponin T *(*TnT*), a pivotal protein in the assembly of skeletal muscles sarcomeres and a modulator of calcium driven muscle contraction, during flatfish metamophosis is studied.

**Results:**

In the present study five cDNAs for halibut *TnT *genes were cloned; three were splice variants arising from a single *fast TnT *(*fTnT*) gene; a fourth encoded a novel teleost specific fTnT-like cDNA (*AfTnT*) expressed exclusively in slow muscle and the fifth encoded the teleost specific *sTnT2*. THs modified the expression of halibut *fTnT *isoforms which changed from predominantly basic to acidic isoforms during natural and T4 induced metamorphosis. In contrast, expression of red muscle specific genes, *AfTnT *and *sTnT2*, did not change during natural metamorphosis or after T4 treatment. Prior to and after metamorphosis no change in the dorso-ventral symmetry or temporal-spatial expression pattern of *TnT *genes and muscle fibre organization occurred in halibut musculature.

**Conclusion:**

Muscle organisation in halibut remains symmetrical even after metamorphosis suggesting TH driven changes are associated with molecular adaptations. We hypothesize that species specific differences in *TnT *gene expression in teleosts underlies different larval muscle developmental programs which better adapts them to the specific ecological constraints.

## Background

*Troponin T *(*TnT*) is a class of skeletal muscle specific proteins that are an important component of the thin-filament. TnT proteins are essential for correct assembly of the sarcomeres [1,2] and are responsible for anchoring of the Troponin complex to tropomyosin (Tm) and correct assembly and function of Troponin I and C [3,4]. In tetrapods, three *TnT *genes exist, fast, slow and cardiac, expressed respectively in white (fast-twitch and glycolytic), red (slow-twitch and oxidative) and cardiac muscle. However, recent studies indicate that in teleosts a greater number of genes exist and at least two *fast TnT *(*fTnT*) and two *slow TnT *(*sTnT*) genes exist as well as an apparently teleost specific *intronless TnT *(*iTnT*) gene [5-7].

In terrestrial vertebrates, TnT genes are known to produce multiple protein isoforms by alternative splicing mechanisms [3,4,6,8-20]. A number of factors, such as contractile properties [21], intracellular pH [22] in myofibres, calcium dependence modulation during cross-bridge cycling [23] and innervation patterns during development [24], are proposed to be associated with TnT isoform switching in cardiac and fast muscle of foetal and adult terrestrial vertebrates. In contrast, in terrestrial vertebrates, no developmental specific sTnT protein isoform changes occur [4,9,13,18-20], although in adults red-muscle-specific isoforms are detected [3].

We recently reported the existence of three stage specific *fTnT *transcripts in a teleost, the sea bream, namely embryonic, larval and adult which are splice variants of a single gene [5]. Moreover, in contrast to tetrapods, two paralogue *sTnT *genes (*sTnT1 *and *sTnT2*) that exhibit developmental specific expression were also identified in sea bream [6]. The teleost specific gene, *sTnT2*, is first detected in sea bream in late epiboly stages and is the only *sTnT *gene expressed up until 4 days post-hatch (dph) the time at which *sTnT1 *expression starts [6]. Throughout larval development and in early juvenile stages, *sTnT1 *is the predominant *sTnT *gene expressed in sea bream red muscle, although in adult red muscle *sTnT2 *is the predominant isoform and *sTnT1 *is virtually undetectable [6].

Studies in flatfish indicate that changes occur in muscle during the thyroid hormone driven metamorphosis in which a bilaterally symmetrical larvae changes to an asymmetric juvenile. In pre-metamorphic pelagic larvae of the flatfish *Paralichthys olivaceus *(flounder) two fTnT immunoreactive proteins of 41.5 and 34 kDa were reported [25]. However, when the larvae enter metamorphosis to become a benthic flatfish, the 41.5 kDa protein is substituted by a new 33.5 kDa isoform. In post-metamorphosis juvenile fish only the 34 and 33.5 kDa isoforms of TnT are present [25]. Similarly, during spontaneous metamorphosis larval 5,5'-dithio-bis-nitrobenzoic acid (DTNB) light chain (myosin light chain 2, MLC2) is replaced by an adult specific isoform [26]. Although it is evident that changes which occur in muscle during metamorphosis are probably associated with changing functional requirements, surprisingly few molecular studies exist of this process. In the present study in order to analyse changes in skeletal muscle and in particular TnT gene expression during halibut (*Hippoglossus hippoglossus*) natural and T4 induced metamorphosis, cDNAs for slow and fast TnT were cloned and their expression and tissue distribution was studied in relation to changing thyroid hormone concentrations and muscle development.

## Results

### Halibut TnT genes

**Figure 1:**
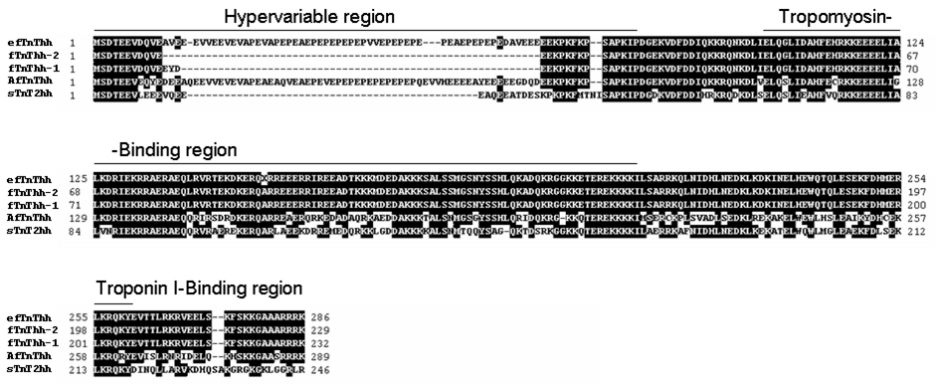
ClustalX multiple protein sequence alignment of halibut predicted TnT protein; Clustal X multiple alignment of predicted protein sequences from halibut *fTnThh *cDNA isoforms, *AfTnThh *and *sTnT2hh *cDNAs. Shaded areas represent sequence similarity. The N-terminal hypervariable region and Tropomyosin- and Troponin I-binding regions are indicated.

Five cDNAs corresponding to different skeletal muscle *TnT *genes (Fig. 1) were isolated from a cDNA library of metamorphosing halibut larvae. In tBLASTx analysis [27] against the GeneBank database, three cDNAs gave a highly significant match with teleost, fTnT genes. From the analysis it was determined that the *fTnThh *cDNAs isolated correspond to a putative embryonic/larval halibut fTnT (denominated *efTnThh*; DQ680173) and two different adult isoforms (denominated *fTnThh-1*, DQ680174, and *fTnThh-2*, DQ680175). The *efTnThh *cDNA is a full-length clone with 965 nucleotides (nt) and encodes a 286 amino acids (aa) protein from nt 62 to 919. The size of the deduced protein is 34.6 kDa and the predicted pI is 5.27 (Fig. 1)[28]. The *fTnThh-1 *isoform is a 752 bp cDNA which encodes a protein of 232 aa from nt 22 to 717 (Fig. 1). The deduced fTnThh-1 protein has a predicted molecular weight of 27.89 kDa and a pI of 9.42 [28]. The cDNA of the third isoform, denominated *fTnThh-2*, is 1,020 bp long and encodes a putative protein of 229 aa from nt 70 to 756 (Fig. 1). The predicted molecular weight and pI for the fTnThh-2 protein isoform is respectively 27.5 kDa and 9.55 [28].

*In silico *characterisation of the deduced halibut fTnT proteins using ProDom [29] and PRINTS [30] software confirmed that they possess all the characteristics of fully functional fTnT proteins. ClustalX [31] multiple alignment analysis of these putative halibut *fTnT *cDNAs and their deduced protein sequence indicates that they are the products of alternative splicing of the halibut *fTnT *gene (Fig. 1). Isoform efTnThh shares 80% sequence identity with fTnThh-1 and -2 isoforms, whereas isoforms fTnThh-1 and -2 are 99% similar. The differences between the halibut *TnT *cDNA isolated are due to the presence of an insert in efTnThh (aa 12 to 68) and fTnThh-1 (aa 12 to 14) which is lacking in the fThThh-2 isoform (Fig. 1).

A further 1,107 bp cDNA was also cloned and tBLASTx analysis [27] suggests that it most closely resembles an *fTnT *gene and gave the most significant hit to *D. rerio fTnTa *gene and it was tentatively designated an *atypical fast TnT *cDNA (*AfTnThh-1*; DQ680176) as a consequence of its tissue distribution. The predicted protein product encoded by *AfTnThh-1 *cDNA was 289 aa with a pI of 5.07 and molecular weight of 34.21 kDa (Fig. 1)[28].

A halibut cDNA homologous to a previously reported teleost specific *sTnT2 *gene was also cloned. This cDNA, designated *sTnT2hh *(DQ680172), is 980 bp long and encodes a deduced protein of 246 aa (Fig. 1) with a predicted molecular weight of 29 kDa and a pI of 9.24 [28]. It was not possible, despite extensive cDNA library screening to isolate a cDNA that was the product of a putative halibut sTnT1 gene.

ClustalX multiple sequence alignment [31] of the deduced amino acid sequence of halibut TnT genes show that efTnThh shares 69% and 52% sequence identity, respectively, with AfTnThh and sTnT2hh. Comparison of the other halibut fTnT-1 and -2 isoforms with AfTnThh and sTnT2hh reveal they share ~59% sequence identity while, AfTnThh and sTnT2hh share 50% identity.

### Putative genomic organization of halibut skeletal TnT genes

**Figure 2:**
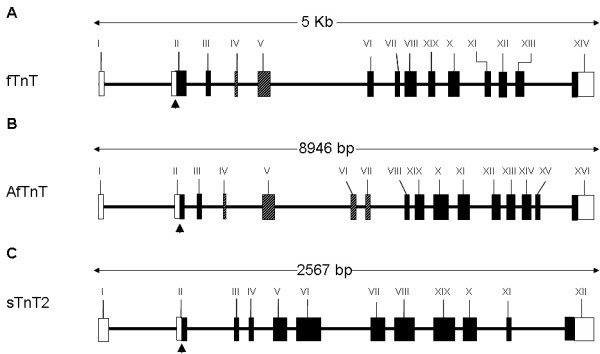
Genomic organization of *Tetraodon nigroviridis TnT *genes; Genomic organization of *Tetraodon fTnTtn *(**A**), *AfTnTtn *(**B**) and *sTnT2tn *(**C**). Open rectangles represent untranslated regions, while shading represents coding exons. The striped rectangles represent alternatively spliced exons. The arrowheads pointing upwards depict the ATG translational start codon. The three *Tetraodon TnT *genes have a very similar genomic organizations with exon I containing the start of the 5'UTR, the ATG start site is located in exon II and the last exon codes for the last C-terminal amino acids as well as the entire 3'UTR. In the *fTnT *gene exons IV and V are alternatively spliced both in *Tetraodon *and halibut. In the *AfTnT *gene exon VI is alternatively spliced in *Tetraodon *but seems to be constitutive in halibut (see Discussion).

*In silico *tBLASTx analysis [27] using halibut *fTnT *cDNA sequences gave a highly significant hit to *Tetraodon nigroviridis *genomic scaffold 7217 (SCAF7217). The putative *Tetraodon *and deduced halibut *fTnT *gene is composed of 14 exons (Fig. 2A) as previously described for the sea bream [5]. The *efTnThh *isoform is composed of exons I to III and V to XIV (Fig. 2A). Isoform *fTnThh-1 *contains all exons except exon V, which codes for the highly acidic peptide containing a glutamic acid (E), proline (P) repeat (Fig. 1 and 2A). In the isoform *fTnThh-2 *mRNA, exons IV and V are spliced out (Fig. 2A) which results in the loss of 9 nt in relation to *fTnThh-1 *(Fig. 1). The ATG transcription start signal is located in exon II and exon I bears the beginning of the 5'UTR (Fig. 2A). The 3'UTR and the end of the protein coding region are located in exon XIV. Overall coverage of the *fTnThh *cDNA sequences was 97% and overall identity between it and the *Tetraodon *fTnT gene sequence was 82%.

The halibut *AfTnThh *gene used in tBLASTx analysis led to the identification of the putative *Tetraodon AfTnT *locus in scaffold 15099 (SCAF15099) as well as five different *Tetraodon AfTnT *cDNA isoforms (numbered 1 to 5) which seem to be the product of alternative splicing (*AfTnTtn-1*, CR696067; *AfTnTtn-2*, CR675364; *AfTnTtn-3*, CR662746; *AfTnTtn-4*, CR727722; *AfTnTtn-5*, CR673164). The *Tetraodon AfTnT *cDNA and the *AfTnThh *cDNA were used to deduce the putative genomic organization of the *AfTnT *gene in *Tetraodon *scaffold 15099 by aligning the cDNAs and genomic sequences in Spidey [32]. The analysis revealed that the *Tetraodon AfTnT *gene (*AfTnTtn*) is composed of 16 exons with conserved intron/exon boundaries spanning 8946 bp in *Tetraodon *scaffold 15099 (Fig. 2B). *Tetraodon AfTnTtn *cDNAs had 100% coverage in the *Tetraodon *genomic sequence of scaffold 15099 and the majority of exons shared 100% nucleotide sequence conservation. The halibut *AfTnThh *cDNA had 100% coverage in the *Tetraodon *genomic sequence of scaffold 15099 and shared 79% overall sequence identity. Exon I bears part of the 5'UTR while exon II contains the remainder and the ATG translation start site. Exon III is composed of 13 nt and constitutes together with exons I and II the N-terminal constitutive exons present in all *Tetraodon AfTnT *cDNAs identified. Exon II and III share 77% sequence identity between the *Tetraodon *genomic sequence and halibut *AfTnThh *cDNA, while exon I shares only 58% identity. Exon IV which codes for 3 acidic residues both in *Tetraodon *and halibut is the first N-terminal alternatively spliced exon in *Tetraodon *cDNAs, and shares 75% sequence identity between *Tetraodon *genomic and halibut *AfTnThh *cDNA sequence. Exon V is the largest alternatively spliced N-terminal exon in *Tetraodon *and is the most divergent between the *Tetraodon *genomic sequence and halibut AfTnT cDNA. In this exon the sequence identity is only 52% and a 41 nt insertion in the third quarter of the *Tetraodon *genomic sequence renders this exon bigger in *Tetraodon *than in halibut. However, the 5' and 3' regions of the *Tetraodon *sequence are well conserved with the corresponding halibut *AfTnT *sequence. Exons VI and VII are highly conserved (94%) between the *Tetraodon *genomic sequence and halibut *AfTnT *cDNA sequence and are alternatively spliced in *Tetraodon*. Exons VIII to XVI encode the C-terminal constitutive region present in all vertebrate *TnT *genes and sequence conservation between the *Tetraodon *and halibut was always greater than 85% and no splice variants of this region were observed in *Tetraodon*. Exon XVI codes for the last 11 amino acid residues of the protein and the entire 3'UTR. Although exon XVI shares only 47% sequence identity between *Tetraodon *and halibut the sequence divergence is in the 3'UTR rather than the coding region (86% sequence identity).

The genomic organization of halibut *sTnT2 *gene in *Tetraodon *was also determined (Fig. 2C). Using the *sTnT2hh *sequence in tBLASTx analysis [27] of the *Tetraodon *genome database [33] a single hit with *Tetraodon *scaffold 15000 (SCF15000) was found. A single cDNA transcript (CR734482) arising from *Tetraodon sTnT2 *gene (SCF15000) was isolated and introduced in Spidey aligning software [32] along with the halibut *sTnT2 *cDNA sequence to determine the putative genomic organization of *sTnT2*. The putative *Tetraodon sTnT *locus is composed of 12 exons and spans 2567 nt in the *Tetraodon *genomic sequence (Fig. 2C). Exon I bears the first three-quarters of the 5'UTR and the beginning of the coding region is located in exon II. Exon XII contains the 3'-end of the coding region as well as the 3'UTR (Fig. 2C). The halibut *sTnT2hh *cDNA sequence had 86% coverage in the *Tetraodon *genomic sequence and shared 82% sequence identity.

### Tissue specificity of halibut TnT genes

**Figure 3:**
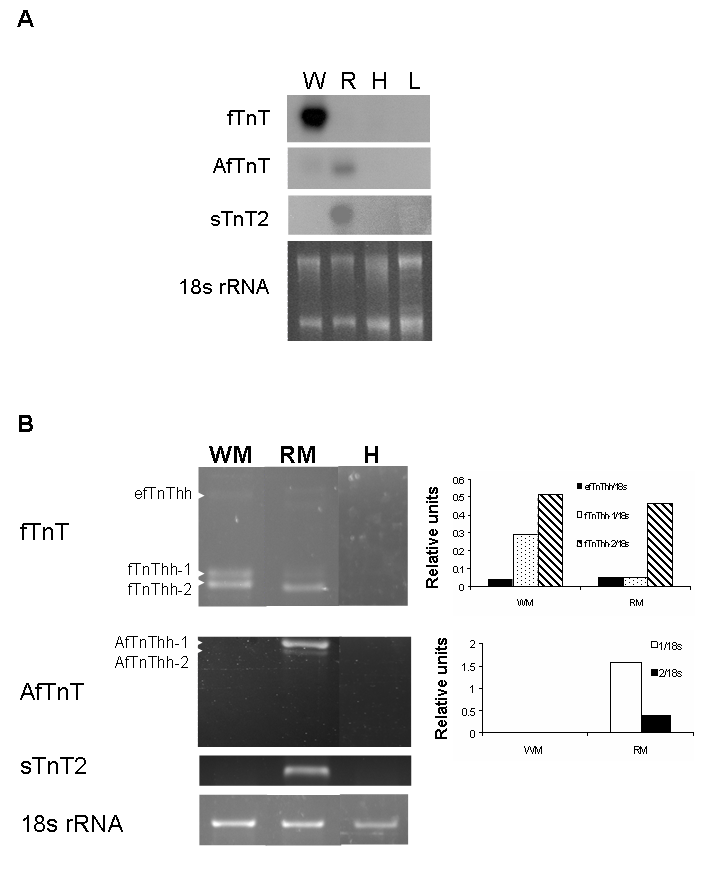
Halibut *TnT *genes tissue specific expression; (**A**) Northern blot analysis of the expression of halibut *fTnThh*, *AfTnThh *and *sTnT2hh *genes in adult white muscle (W), red muscle (R), heart (H) and liver (L). All images represent 48 hour exposures. Ethidium bromide gel image is shown to give an indication of the quantity of total RNA loaded per sample. (**B**) RT-PCR analysis of expression of *fTnThh*, *AfTnThh *and *sTnT2hh *in halibut adult white (WM) and red (RM) muscle and heart (H). The 18s rRNA is shown and was used for normalisation. The graphs on the right of the fTnThh and AfTnThh gels represent, respectively, expression of the different fTnThh or AfTnThh isoforms relative to 18s rRNA.

Northern blot and RT-PCR analysis were carried out in order to determine tissue specificity of the halibut *TnT *genes isolated. The northern blot results (Fig. 3A) show that *fTnThh*, as expected, is expressed in adult halibut white (fast) muscle and is absent from red muscle, cardiac muscle and liver. However, the more sensitive RT-PCR technique revealed that *fTnThh *is also expressed in halibut adult red muscle (Fig. 3B). The tBLASTx and phylogenetic analysis of *AfTnThh *classified this cDNA as the product of a fast TnT gene, although its expression is red (slow) muscle specific (Fig. 3A) and it is not detected in adult white (fast) muscle, cardiac muscle or liver. The halibut *sTnT2 *gene is exclusively expressed in halibut adult red muscle (Fig. 3A). The red muscle tissue specificity of *AfTnThh *and *sTnT2hh *was further confirmed by RT-PCR (Fig. 3B).

### Phylogenetic analysis of Halibut TnT genes

ClustalX multiple sequence alignment [31] of the deduced protein of halibut TnT and other vertebrate TnT protein sequences and striated muscle TnT from *C. elegans *was performed and the resulting phylogenetic relationships were determined in PAUP* version 4.0b software [34] using the maximum-parsimony method with 1000 bootstraps [35] and *C. elegans *TnT as an outgroup.

**Figure 4:**
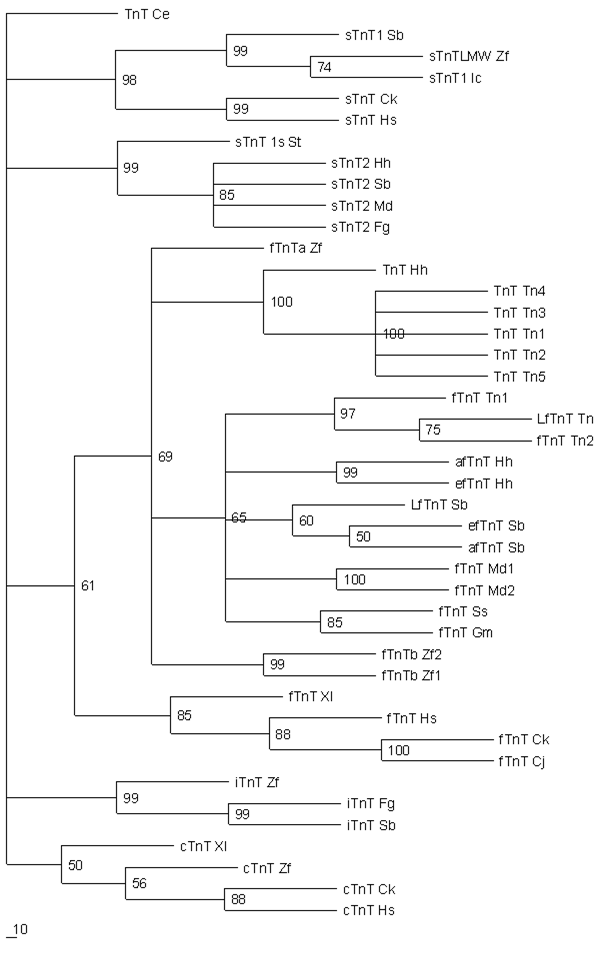
Phylogenetic analysis of halibut *TnT *genes isolated; Maximum parsimony phylogenetic tree using the predicted protein sequence of the different halibut *TnT *cDNA isolated and that of other vertebrates (Table 1) retrieved from Genebank and the Medaka EST database (see Materials and Methods). The bar in the bottom left-hand corner represents 10% sequence divergence. The species and gene abbreviations are described in Table 1.

The phylogenetic tree shows that the vertebrate fTnTs form a single clade and that within it the tetrapod fTnTs cluster apart from the fish fTnTs (Figure 4). Moreover, *efTnThh*, *fTnThh-1 *and *fTnThh-2 *cluster with highly significant bootstrap values with other teleost *fTnT *genes. Within the main *fTnT *clade, *AfTnThh *and *Tetraodon AfTnTtn *isoforms clustered together and formed a separate group and this topology was supported by highly significant bootstrap values (Fig. 4).

Two principal clades were found for *sTnT *which corresponded to *sTnT1 *and *sTnT2 *(Fig. 4). The halibut *sTnT2hh *clusters with other teleost specific *sTnT2 *genes (Fig. 4) and forms a group apart from tetrapod *sTnT *and *sTnT1*, which clustered together.

### *TnT *expression during halibut metamorphosis

**Figure 5:**
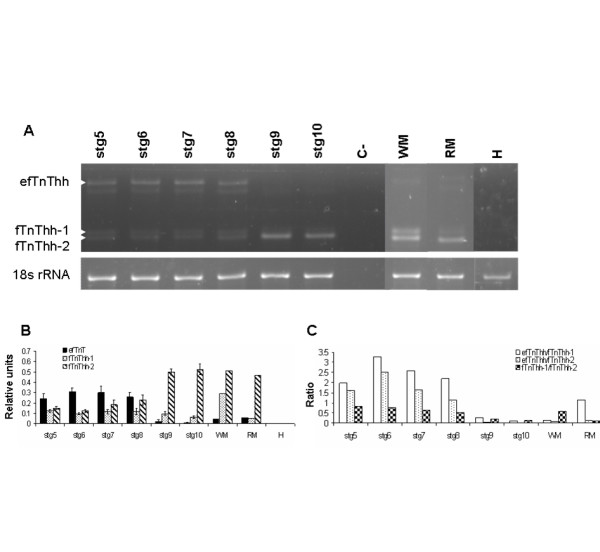
Expression of *fTnThh *gene during halibut metamorphosis; Ethidium bromide gel image of RT-PCR amplified *fTnThh *and *18s rRNA *(**A**). White arrowheads indicate different *fTnThh *isoforms. Graphs represent (**B**) *fTnThh *expression relative to *18s rRNA *and (**C**) the ratio between the different *fTnThh *isoforms. C- indicates the no-template control.

**Figure 6:**
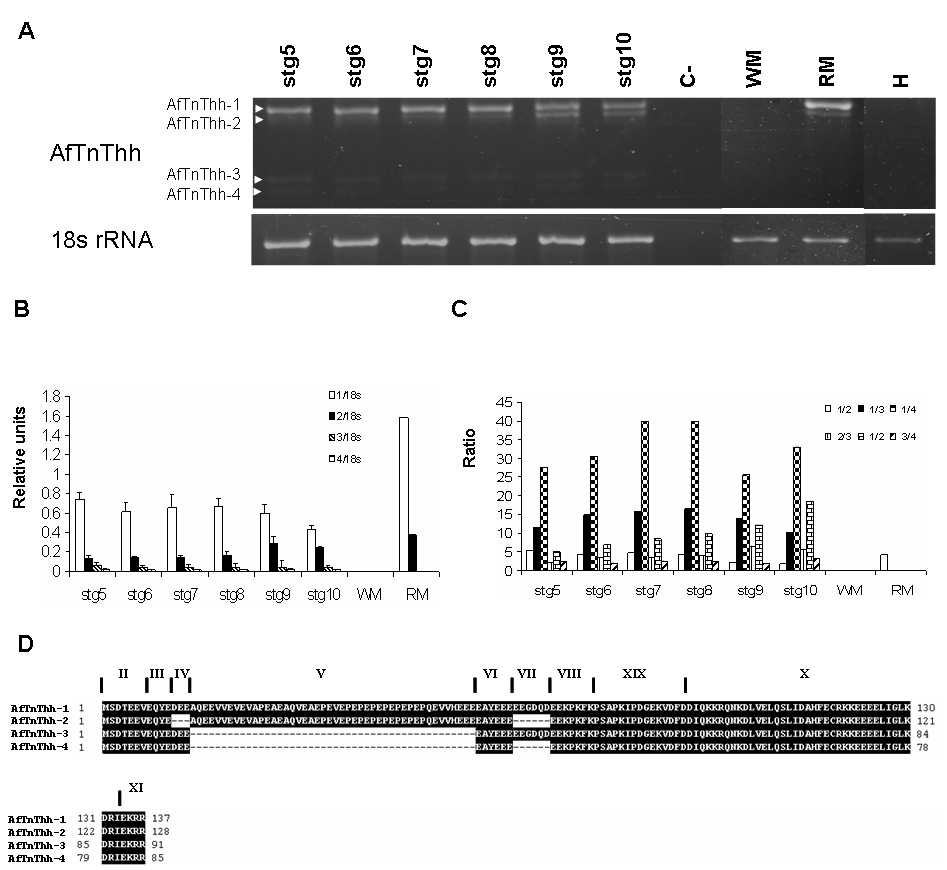
Expression of *AfTnThh *gene during halibut metamorphosis; Ethidium bromide gel image of *AfTnThh *and *18s rRNA *(**A**). C- represents none-template control. White arrowheads indicate the different *AfTnThh *isoforms found. Graphs present the expression of the different *AfTnThh *isoforms relative to *18s rRNA***(B) **and the ratio between the different *AfTnThh *isoforms (**C**). The predicted protein sequence of the amplified halibut *AfTnThh *isoforms is given (**D**). The exons that encode each peptide are also indicated.

**Figure 7:**
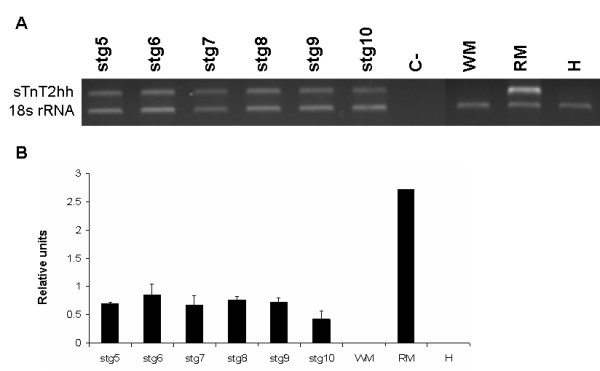
Expression of *sTnT2hh *gene during halibut metamorphosis; Ethidium bromide gel image of *sTnT2hh *and *18s rRNA *expression during halibut metamorphosis (**A**). Graphs present the relative expression of *sTnThh2 *after normalisation with *18s rRNA *during halibut metamorphosis (**B**). C- represents non-template control.

The expression of the isolated halibut *fTnT*, *AfTnT *and *sTnT2 *genes was analysed by RT-PCR during halibut metamorphosis (Fig. 5, 6 and 7, respectively). The primers used in this analysis encompass the entire N-terminal hypervariable region which is known in vertebrates to generate alternative spliced isoforms [3,4,6,8-20]. Three *fTnThh *isoforms were amplified by RT-PCR during halibut metamorphosis, the largest product corresponded to *efTnThh *(423 bp) while the smaller products correspond to, *fTnThh-1 *(261 bp) and *fTnThh-2 *(252 bp; Fig. 5A). A further reaction product is observed between the *efTnThh *and the other two isoforms that does not represent an authentic isoform but a heteroduplex of *efTnThh *and the other two isoforms since isolation followed by PCR, subcloning and sequencing always yield either *efTnThh *or one of the other two isoforms.

The three halibut *fTnT *isoforms are all present in pre-metamorphic halibut larvae (stg 5–7) and *efTnThh *is the predominant isoform expressed up until the beginning of climax and has approximately 2-fold higher expression than *fTnThh-1 *and *-2 *(stg 8; HSD, p < 0.001; Fig. 5A and 5B). At the beginning of climax *fTnThh-2 *expression starts to increase and at the climax of metamorphosis (stg 9) the *efTnThh *isoform is downregulated and is almost undetectable in fully metamorphosed juveniles (stg 10, Fig. 5A and 5B). In parallel *fTnThh-2 *becomes the most highly expressed *fTnThh *isoform and increases ~3-fold in juveniles (Fig. 5A and 5B). Prior to, and after metamorphosis *fTnThh-1 *expression does not change significantly (Fig. 5A and 5B). Analysis of *fTnT *isoform expression in adult halibut muscle by RT-PCR confirmed the general pattern encountered by Northern blot (Fig. 3A). However, the more sensitive RT-PCR technique permitted detection of *fTnThh *isoforms in halibut adult red -muscle (Fig. 3B and 3C). In halibut adult white muscle low expression of *efTnThh *(~14-fold lower than *fTnThh-2*) is observed, *fTnThh-2 *continues to be the predominant isoform although *fTnThh-1 *expression has increased and is about half that of *fTnThh-2 *(Fig. 3B and 3C). The ratio of the different *fTnThh *mRNA in red muscle differs from white muscle since *fTnThh-2 *is ~10-fold more expressed than *efTnThh *and *fTnThh-1 *which are almost undetectable (Fig. 3B and 3C).

**Figure 8:**
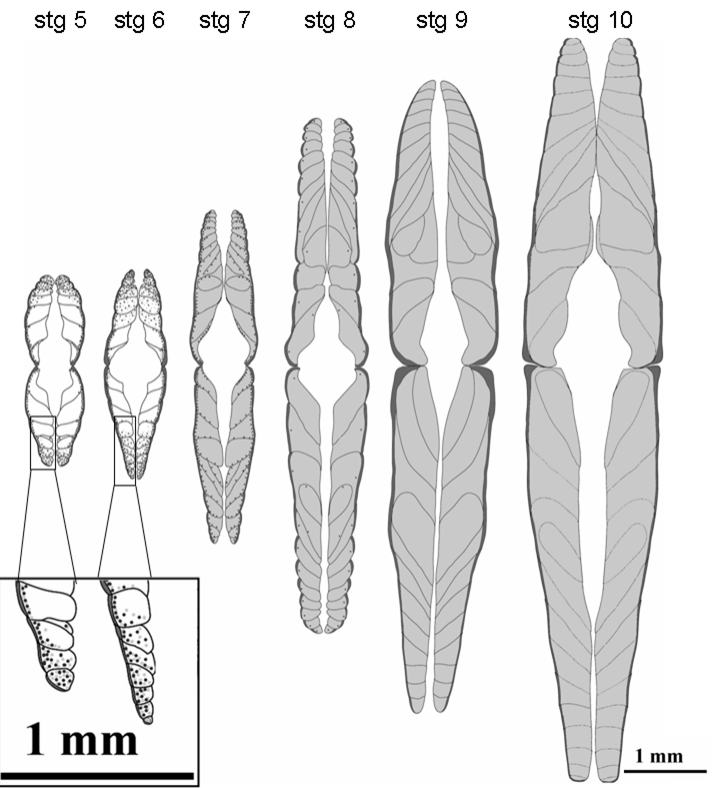
Schematic representation of muscle ontogeny in halibut during metamorphosis; Schematic representation of the muscle ontogeny in halibut from larvae (stg 5) to juvenile (stg 10) and the overall expression pattern of halibut *TnT *genes. There is a general increase in myotome volume during metamorphosis. Note that throughout muscle ontogeny, prior to and after metamorphosis, symmetry is maintained both in the sagital and longitudinal planes. In pre-metamorphic halibut (stg 5 and 6) white muscle hyperplasic small diameter fibers are located in the most apical and lateral sides of the myotome regions (insert) which predominantly express *efTnThh *(black dots). As white muscle fibers increase in size the expression of *fTnThh *isoforms disappears. The expression of the other *fTnThh *isoforms (light grey dots) co-localizes with *efTnThh *expression up until stg 7. When the animals reach the beginning of metamorphosis (stg 7) all mature white muscle fibers express *fTnThh-1 *and *-2 *isoforms (light grey shading) and *efTnThh *is only found in smaller diameter hyperplasic fibers in the periphery of the myotomes around the myosepta (black dots). As the animals enter the climax of metamorphosis expression of the low molecular weight *fTnThh *isoforms is located in the entire myotome (light grey shading) and *efTnThh *is confined to scattered presumptive satellite cells (black dots). At climax (stg 9) and in juvenile (stg 10) white muscle *fTnThh-1 *and *-2 *isoforms are expressed in all the myotome with varying intensity and in fibers close to the myosepta expression is higher. In pre-metamorphic halibut red muscle (dark grey) all cells express *sTnT2hh *and *AfTnThh*. The expression of the red muscle specific *Troponin T *genes is constant and restricted to the outer red muscle throughout halibut metamorphosis and in juvenile animals.

In contrast to *fTnThh*, expression of halibut *AfTnThh *(Fig. 6A) and *sTnT2hh *(Fig. 7A) detected by RT-PCR does not change during metamorphosis (Fig. 7). However, 4 alternatively spliced isoforms of *AfTnThh *are detected (Fig. 8A and 8D). Sequence analysis reveals that the *AfTnThh *isoforms are a result of alternative splicing of exons IV, V and VII (Fig. 2) and are designated *AfTnThh-1 *to *-4*. The largest product, *AfTnThh-1 *(439 bp) is identical to the *AfTnThh *cDNA isolated by library screening and includes all alternatively spliced exons (Fig. 6A and 6D); *AfTnThh-2 *(412 bp; DQ680177) lacks exons IV and VII but exon V is maintained (Fig. 6A and 6D); *AfTnThh-3 *(301 bp; DQ680178) is composed of exon IV and VII but exon V is spliced out (Fig. 6A and 6D); and in *AfTnThh-4 *(283 bp; DQ680179) only exon IV is spliced in and all other alternatively spliced exons (exons V and VII) are spliced out (Fig. 6A and 6D). All the halibut *AfTnThh *isoforms detected contain exon VI (Fig. 6D) and neither the isoform expression pattern nor the ratio between these *AfTnThh *isoforms is altered during metamorphosis (Fig. 6A–C). A small but statistically significant (HSD, p ≤ 0.005; Fig. 6A and 6B) decrease in expression of all the isoforms is observed in stg 10 juveniles; *AfTnThh-1 *is the predominant isoform throughout the halibut's life. In turn, *AfTnThh-2 *is the second most abundant isoform and its expression increases after climax (HSD, p ≤ 0.006; Fig. 6A and 6B). However, in adult red muscle the ratio of the two isoforms is identical to pre-metamorphic stg 5 animals (Fig. 6A and 6C). The *AfTnThh-3 *and *-4 *isoforms have identical (HSD, p > 0.05), very low expression (Fig. 6A–C) and their expression and ratio in relation to other isoforms does not change during metamorphosis (HSD, p > 0.05; Fig. 6A–C). Moreover, in adult red muscle these low molecular weight isoforms are not expressed (Fig. 6A and 6B).

### Spatial-temporal expression pattern of halibut *TnT *genes during metamorphosis

The spatial-temporal expression pattern of *TnT *mRNA was determined in free- swimming halibut larvae which feed exogenously. In such larvae two distinct muscle layers are evident, an inner white and superficial red muscle layer (Fig. 8). In stage 5 halibut the anatomical organisation of the white muscle is very simple with a "*V*" shaped myomere and it is composed of several block-like bilaterally symmetrical myotomes bounded by a septum. The extreme dorsal and ventral myotomes and the lateral region of the myotomes close to the red muscle is characterized by the presence of numerous small rounded fibres, characteristic of germinal zones, while in the deeper region of the larger myotomes, fibres are much larger, more block-like and are closely packed. In subsequent stages myotome number increases and in stage 6 halibut larvae they take on the typical "*W*" shaped myomer organisation.

No change in the bilateral symmetry of the muscle anatomy accompanies metamorphosis, although a significant increase in the volume of muscle occurs in stages 8, 9 and 10 (Fig. 8). Regions of hyperplastic growth persist in the most ventral and dorsal myotomes in all of the stages analysed. A rapid increase in myotome number is evident from stage 5 through 7 and in subsequent stages hypertrophic growth leads to a considerable increase in myotome and overall muscle volume. In stage 5 the red muscle layer is a monolayer of fibres at the outermost region of the myotomes, although proliferating red muscle fibres at the midline region of the myotome generate a small region with a double layer of fibres. In stage 6 larvae this double layer is much more evident and in successive larval stages all the red muscle layer is proliferating so that in stage 9 halibut it is composed of several layers of fibres of different sizes. Notably during the entire metamorphic process the symmetry of the myotomes is maintained even though the skull becomes asymmetric with the right eye migrating to the left side of the head that at the end of metamorphosis constitutes the dorsal side of halibut body.

**Figure 9:**
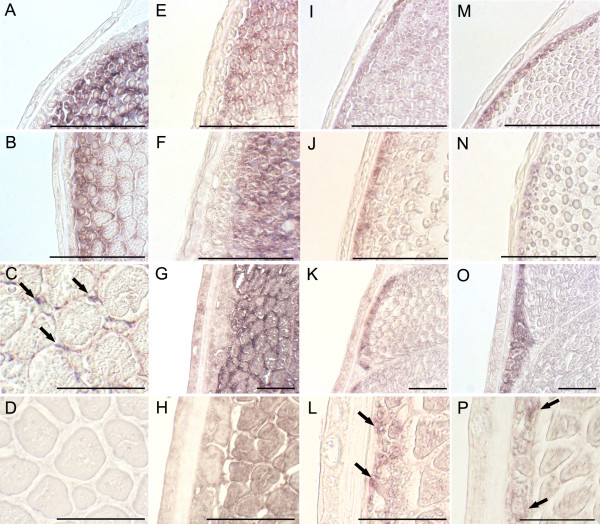
In situ expression of halibut *TnT *genes during metamorphosis; Temporal and spatial expression of halibut embryonic/larval *fTnT *exon (A-D), General 3'UTR-*fTnT *probe (E-H), *AfTnT *(I-L) and *sTnT2 *(M-P) in transversal sections of halibut larvae, by in situ hybridization using DIG labelled riboprobes. Pre-metamorphic stg 5 larvae: A, E, I, M; pre-metamorphic stg 6 larvae: B, F, J and N; larvae at the beginning of metamorphic climax (stg 8): C, G, K and O; larvae at metamorphic climax (stg 9): D; postmetamorphic juveniles (stg 10): H, L and P. In C arrows depict *efTnThh *expression in putative myogenic satellite cells. In L arrows depicts *AfTnT *expression. In P arrows depict *sTnT2 *expression. Scale bars, 50 μm.

Halibut embryonic/larval *fTnT *exon expression in pre-metamorphic halibut larvae (Stg5 and 6; Fig. 8 and Fig. 9A and 9B) is restricted to white muscle, in the lateral and apical germinal zones of the myotome. As the halibut larvae approach metamorphic climax (Stg8; Fig. 8 and Fig. 9C) the *efTnThh *expression is significantly downregulated and confined to very small fibres and to presumptive satellite cells (arrows in Fig. 8 and Fig. 9C). From climax of metamorphosis (stg 9; Fig. 8 and 9D) onwards no *efTnThh *expression is detected. The 3'UTR *fTnT *probe reveals the general *fTnThh *expression pattern in stage 5 (Fig. 8 and Fig. 9E) and stage 6 larvae (Fig. 8 and Fig. 9F) which is similar to that observed with the embryonic/larval *fTnT *exon probe. At stage 8 (Fig. 8 and Fig. 9G) *fTnT *is uniformly distributed in the white muscle. As the animals reach post-metamorphic juvenile stage 10 (Fig. 8 and Fig. 9H) *fTnThh *expression looses its uniformity and although still expressed in the entire myotome, the signal is more intense in cells close to the myosepta (Fig. 8).

Halibut *AfTnT *and *sTnT2 *gene expression is confined to red muscle fibres in all metamorphic stages analysed. Expression of *AfTnT *in pre-metamorphic stage 5 larvae (Fig. 8 and Fig. 9I) is highly abundant in the red muscle layer. At the end of larval life, just before the onset of metamorphosis, (stg 6) in the myotome midline region two red muscle cell layers both expressing *AfTnT *are present (data not shown). In stage 8 (Fig. 9K) the expression of *AfTnThh *is detected uniformly in all the fibres of the red muscle, while in post-metamorphic juveniles at stage 10 (Fig. 8 and Fig. 9L) it is mostly present in the smaller fibres (arrowheads in Fig. 9L). In the case of sTnT2, expression is restricted to the red muscle layer and is very similar to *AfTnT *in stage 5 (Fig. 9M), 6 (Fig. 9N), 8 (Fig. 9O), 10 (Fig. 9P) halibut. The spatial-temporal expression pattern of red muscle specific halibut TnT genes is symmetrical and does not change during halibut metamorphosis (Fig. 8 and 9).

### TH levels in metamorphosing halibut

**Figure 10:**
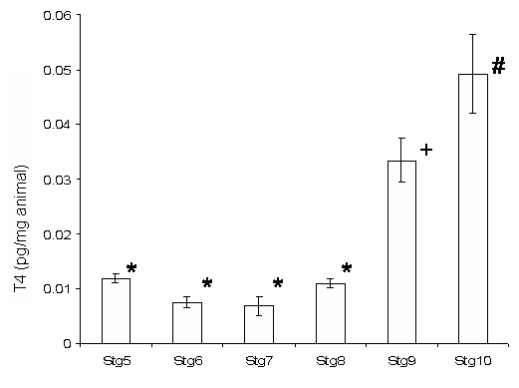
Halibut whole-body T4 levels during metamorphosis; Change in whole-animal T4 content (pg/mg) during halibut metamorphosis. *- No significant statistical difference (HSD, p > 0.05). + and # denote significant statistical differences from all other time points (HSD, p < 0.05).

At the beginning of metamorphosis and up until the beginning of climax (stg 8), T4 levels remain low with no significant differences observed between stages 5, 6 and 8 (HSM, p > 0.05; Fig. 10). In fact in larvae at the beginning of metamorphosis (stg 7) T4 levels are lower than in pre-metamorphic larvae (stg 5, HSD, p < 0.05). At the climax of metamorphosis (stg 9), whole-body T4 content increases about 3-fold in relation to all the previous stages (HSM, p < 0.005, Fig. 10). In post-metamorphic juveniles (stg 10) T4 levels continue high and, although not significantly different from stage 9, they are significantly higher than all other preceding stages (stg 5 to 8, HSM, p < 0.001; Fig. 10).

Before metamorphosis and up until climax of metamorphosis (stg 9) T3 levels are lower than T4 (HSD, p < 0.001; Fig. 10). In fact, T3 levels decrease from pre-metamorphic larvae (stg 5) until the start of metamorphosis (stg 8) (Fig. 10). However, in halibut at climax (stg 9) T3 levels increase more than 200-fold and are higher than T4 levels (HSD, p < 0.001; Fig. 10). However, after metamorphosis T4 levels are again higher than T3 levels (HSD, p < 0.001; Fig. 10). T3 levels at climax and in fully metamorphosed juveniles are significantly higher than all previous halibut stages (HSD, p < 0.001; Fig. 10).

### T4 treatment and halibut TnT expression

**Figure 11:**
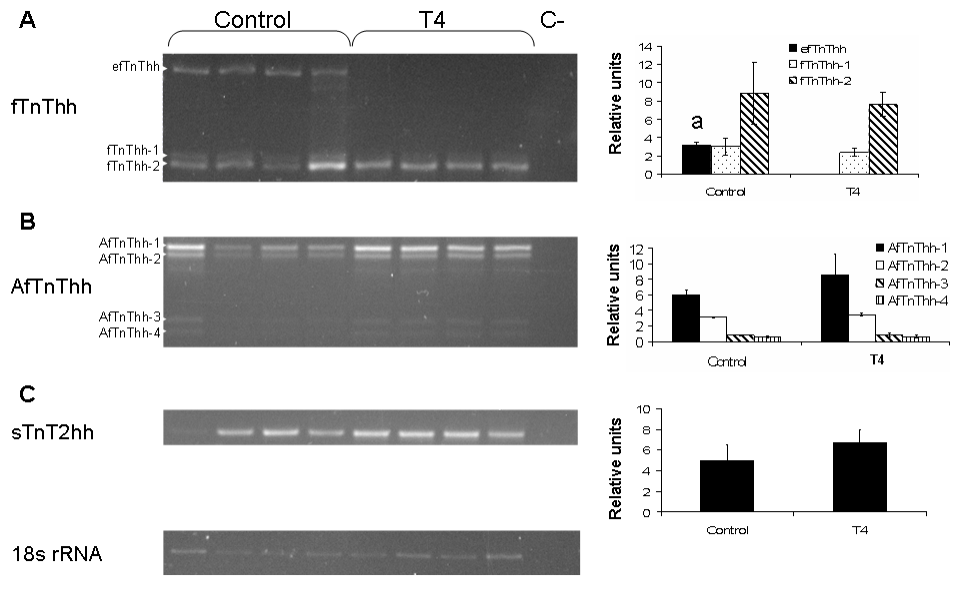
Effect of T4 treatment on halibut *TnT *genes expression in pre-metamorphic larvae; Ethidium bromide gels showing expression of *fTnThh *(**A**), *AfTnThh *(**B**), *sTnT2hh *(**C**) and *18s rRNA *genes in pre-metamorphic larvae treated for 10 days with T4 or untreated (control). Graphs on the right side of gel images represent *fTnThh *and *AfTnThh *isoform expression and *sTnT2hh *expression in relation of *18s rRNA *in control and T4 treated pre-metamorphic (stg 5) halibut larvae for 10 days. *- represents significant statistical differences between *efTnThh *expression in control and T4 treated halibut larvae (HSD, p < 0.05). No reference signifies no significant statistical difference (HSD, p > 0.05).

Treatment of pre-metamorphic halibut larvae for 10 days with T4 totally repressed *efTnThh *isoform expression (HSD, p < 0.05, Fig. 11A) without affecting the expression of the other two fTnThh isoforms (HSD, p > 0.05, Fig. 11A). Neither *AfTnThh *or *sTnT2hh *expression or isoform profile is altered from that of control larvae by T4 treatment (Fig 11B and 11C).

## Discussion

We have isolated cDNAs from three different striated muscle TnT genes in the halibut one of which is white muscle specific while the other two are red muscle specific. Alternative splicing of the *fTnThh *gene gives rise to three alternative splice variants which encode three proteins varying only in the N-terminal region (Fig. 1). As observed for the fTnT gene of *S. auratus *[5], halibut *fTnT *is also expressed in adult red muscle. In fact, the expression of teleost fast-specific genes in red muscle seems to be a common feature in teleost species [36,37]. In relation to fTnThh-1 (predicted M_R _27.89 kDa and pI 9.42) and fTnThh-2 (predicted M_R _27.5 kDa and pI 9.55), the putative efTnThh protein isoform is bigger (M_R _34.6 kDa) and more acidic (pI 5.27) and the difference arises as a consequence of splicing in of exon V (Fig. 2A), in a way similar to that previously reported in *S. auratus*, a perciform and also in other vertebrates [4,10-13], [38-50]. In the halibut the number of alternatively spliced *fTnT *isoforms are the same as found in *Tetraodon *and *S. auratus *[5]. In common with *Tetraodon efTnT*, exon V is spliced in and exon IV is spliced out (Fig. 1 and 2A). This is in contrast to *efTnT *in *S. auratus *in which exon IV is also spliced in along with exon V [5] suggesting that in the halibut and *Tetraodon *exons IV and V are mutually exclusively expressed (Fig. 2A). Although no data is available about the specific biochemical characteristics of muscle fibres in halibut, *S. aurata *or *Tetraodon *larvae, the present study indicates that differences probably exist between teleost species and these differences are probably an adaptation to their differing ecologies and locomotive strategies.

In rat, human and mouse it has been reported that some fTnT isoforms predominate over others due to the specific biochemical characteristics of different fibres types [51]. In these mammals acidic, fetal fTnT isoforms predominate in mainly glycolytic fibres whereas more basic isoforms are predominant in mainly oxidative fibres [51]. However, there are clear differences between teleost and tetrapod *fTnT *genes. In tetrapods alternative splicing of the fTnT gene generates several N-terminal protein isoforms through the use of a greater number of alternatively spliced exons [4,11] whereas in teleosts it seems that only two exons undergo alternative splicing (Fig. 2A; [5]). Although the fetal/embryonic exon is bigger in teleosts than in tetrapods they have similar biochemical characteristics as it encodes an acidic peptide containing several glutamic acid residues (present study; [5,12]). No *fTnT *cDNA isoforms which give rise to 3' spliced variants and therefore proteins with differing C-terminal sequences are observed in halibut, *Tetraodon *or *S. auratus*. Curiously, the deduced C-terminal amino acid sequence of all halibut *fTnT *isoforms share greatest identity to tetrapod isoforms containing the embryonic specific exon 17 [4,10,47,52]. Together with a previous study in teleosts [5] the present data in halibut seem to reinforce the idea that the occurrence of alternatively spliced exons in the C-terminal region of the *fTnT *gene is a characteristic exclusive to terrestrial vertebrates. Nonetheless, the genomic organization of *fTnT *genes in teleosts is identical to tetrapods [11] and from an evolutionary perspective, it seems likely that alternative splicing to generate N-terminal protein variants of the *fTnT *gene in vertebrates already occurred before the divergence of actinoperigii and sarcopterigii vertebrate lineages.

An unexpected observation arising from the present study was the identification of a large (predicted M_R _34.21 kDa), highly acidic (predicted pI 5.07) halibut skeletal muscle *TnT *(AfTnT) gene with high sequence identity to *D. rerio fTnTa *gene [7]. A homologue of this TnT gene was found in *Tetraodon *together with five splice variants and despite its unusual characteristics it had a similar organization to other vertebrate *TnT *genes [4,7,10-13,19,38-52]. The highly acidic nature of the protein isoforms encoded by this gene is a consequence of splicing in of exon V which encodes a very acidic stretch (~50 aa) of amino acids. Regardless of the fact that both sequence similarity and phylogenetic analysis categorise the putative AfTnThh protein as a fast TnT the tissue expression analysis (Fig. 3) indicates that the *AfTnT *is a red muscle specific gene in halibut. This is the first time to our knowledge that a gene presumed to be fast muscle specific in vertebrates is exclusively expressed in red muscle. The observations of *TnTs *in halibut and zebrafish [7] indicate that despite the apparent anatomical simplicity of striated skeletal muscle in teleosts, at the molecular level they have novel adaptations that probably underline species-specific control mechanisms of muscle development.

### Halibut *TnT *genes expression during metamorphosis

The expression of *AfTnThh *and *sTnT2hh *transcripts does not appear to change at climax of metamorphosis, although changes in the expression of fTnT gene isoforms do coincide with this developmental stage in halibut. The transition in expression of *fTnT *isoforms is correlated with the increase in whole-body T3 and T4 levels and the downregulation of *efTnThh *(Fig. 5) In turn, TH levels correlate positively with the increase in expression of *fTnThh-2 *(Fig. 5, 9 and 10). A similar situation also occurs in other flatfish, *Paralichthys olivaceus *[25], *Solea solea *and *Scophthalmus maximus *[53] and at the climax of metamorphosis an acidic efTnT isoform is downregulated and lower molecular weight and more basic isoforms are upregulated, and detected in juvenile and adult white muscle.

The changes in *fTnT *isoform expression in different teleost (present study, [5,25,53]) are reminiscent of what occurs in tetrapods in which fetal acidic *fTnT *isoforms are downregulated immediately after birth and are substituted by basic adult isoforms [10,54,55]. Moreover, a common mechanism of alternative splicing of 5' exons in the fTnT gene in teleosts, birds and mammals, implied by similar codon splitage combinations [5,11], further reinforces the hypothesis that alternative splicing of the fTnT gene is conserved and occurs in order to cope with similar developmental demands on muscle in all vertebrates. The transition from acidic to a basic isoforms in *fTnT *genes seems to be a common trend in vertebrates and represents a physiological, mechanistic and functional adaptation of developing striated muscle [3,10,22,23]. This strong conservation suggests that the same factors responsible for *fTnT *isoform transition during development are common throughout vertebrates and may be a trait acquired long before the divergence of the actinoperigii and sarcopterigii vertebrate lineages. In fact, teleost muscle and tetrapod muscle face similar physiological and biochemical changes during development. Foetal mammalian muscle grows by hyperplasia up until birth and muscle fibres are mainly glycolytic while the mainly oxidative adult muscle fibres, that differentiate after birth, express predominantly basic fTnT isoforms [51]. In teleosts, the muscle fibres also change their biochemical and physiological characteristics during development and up until metamorphosis white muscle is the major respiratory surface of the larvae and fibres are mainly aerobic and rich in mitochondria whereas the adult muscle fibres are mainly anaerobic [56-62].

As shown by others, and in contrast to tetrapod post-embryonic muscle development, in teleost species of large size, like the halibut, post-embryonic muscle development takes place in two steps in which hyperplasia is the main mechanism of muscle growth [36,56,58,60,61,63,64]. The first hyperplasic phase of post-embryonic muscle development in large size teleost larvae is characterised by proliferative epaxial and lateral areas of the myotome and as animals reach the juvenile stage these regions are depleted and a second stage of hyperplasic growth continues in scattered myogenic cells throughout the myotome. Notably, the *efTnThh *isoform is mainly found at the most epaxial and lateral zones of halibut pre-metamorphic larval white muscle myotome, especially in small diameter white muscle fibres (Fig. 8 and 9A–E and A'-E'). The early differentiated halibut larvae white-muscle myoblasts appear to first express predominantly *efTnT *and as they mature and are incorporated in the myotome expression is downregulated and other *fTnT *isoforms take their place. In vertebrates the embryonic to adult *fTnT *isoforms pattern of change is related to alterations in cellular pH, metabolic and physiological characteristics of maturing muscle fibres [3,10,22,23]. The transition from acid to basic pI fTnT isoforms in developing halibut muscle may be associated with the transition from proliferative small diameter muscle cells to more basic larger white muscle fibres. In fact, in *S. auratus *it was found that larval small diameter myoblast cells located in the hyperplasic lateral and epaxial region of the larval myotome contain acid mATPase activity whereas mature large diameter muscle fibres have mild alkali mATPase activity [36]. Together with the downregulation of expression of *efTnThh *at halibut metamorphosis these hyperplasic proliferative areas of the myotome are gradually depleted just as metamorphosis starts and totally absent in animals entering climax (Fig. 8 and 9A–E and A'-E'). This resembles the situation in *S. auratus *where at the end of larval life the same lateral and apical hyperplasic white muscle germinative areas are depleted [65]. However, and in contrast to what occurs in *S. auratus *[5], the treatment of pre-metamorphic halibut larvae with T4 shows that thyroid hormones control the expression profile of fTnT isoforms in the halibut and also in other flatfish [25].

The relationship between THs and change in fTnT isoform expression has yet to be directly demonstrated in teleosts. It is notable that despite the accepted role of THs in driving flatfish metamorphosis and the change from a symmetrical larvae to an asymmetrical juvenile, the way in which muscle symmetry changes has been largely ignored. In the present study muscle development was found to be a bilaterally symmetrical process before, during and after metamorphosis as revealed by histology and *in situ *gene expression studies. Nonetheless, the changes in TnT isoform expression in halibut, *S. solea*, *S. maximus *and *P. olivaceus *indicate muscle is TH responsive [53,25] as has been demonstrated in mammals. In rats T3 increased the expression of Ca^2+ ^ATPase specifically in white muscle fibres and the increase in relaxation rate of post-embryonic white muscle was strictly dependent on THs [66]. Moreover, in mammals THs are necessary to complete development of skeletal muscle [67]. The results from experiments in teleosts suggest that various post-embryonic muscle development mechanisms exist. For example, in *S. auratus *[5] slow muscle seems to be more sensitive to THs than white muscle, whereas in halibut, expression of the slow-muscle specific genes *sTnT2hh *and *AfTnThh *does not alter during metamorphosis (when endogenous T4 levels rise) or in response to exongenous T4 (Fig. 6, 7 and 11).

## Conclusion

Together with previous studies in teleost *TnT *genes [6,7,25,53] the present work shows that teleost muscle, although apparently simpler and having a smaller number of specialized muscles in comparison to tetrapods [3,4] shows remarkable genetic heterogeneity and species-specific regulation. The diversity of *TnT *forms in teleost muscle arise from alternative splicing but also from a new teleost specific *TnT *gene and this heterogeneity probably contributes to better adapt the musculature to the specific functional demands of different teleost species. In common with other flatfish, but in contrast to the round fish, *S. auratus *[5], the halibut *fTnT *gene isoform expression profile is regulated at metamorphosis by THs, although halibut red muscle specific genes seem to be insensitive. Interestingly, the asymmetry arising during flatfish metamorphosis does not extend to the musculature and the temporal and spatial expression patterns of *TnT *genes and muscle fibre organization remains symmetrical before, during and after metamorphosis (Fig. 8 and 9). Clearly THs regulate muscle development but far more work is required to establish the specific molecular and cellular events during flatfish and round fish muscle development.

## Methods

### TnT cDNA library screening

A lambda phage cDNA library made from metamorphosing larvae of halibut was plated in densities ranging from 1,000–5,000 plaque forming units (pfu). A probe was obtained for skeletal TnT by randomly isolating and sequencing 10 clones. A putative halibut *fTnT *obtained in this way was used as a probe for cDNA library screening or alternatively a *PstI*/*EcoRI *digested cDNA fragment from a sea bream *sTnT2 *gene (*sTnT2sb*)[6] was utilised. In each screen using the halibut fTnT or the *sTnT2sb *probe, nitrocellulose membrane lifts were performed and membranes pre-hybridized for 2 hours, respectively, at 65°C or 60°C in hybridization solution (6×SSC, 0.1%SDS, 100 μg/mL tRNA, 5× Denhardt's). DNA probes were labelled with [^32^P] by random priming (Megaprime, random labelling kit, Amersham Biosciences, UK) and purified on a sephadex column. Radioactively labelled probes were diluted in new hybridization mix and allowed to hybridize overnight with the membranes. Two post-hybridisation stringency washes were carried out for ~30 minutes at room temperature (1×SSC, 0.1%SDS) followed by two 30 minute washes (1×SSC, 0.1%SDS) at 65°C or 60°C, respectively. Membranes where then exposed overnight at -80°C to Biomax MS film (Kodak, Palo Alto, CA, USA) and several positive plaques were isolated and automatically excised into pBluescript SK+/- (Stratagene), DNA purified and cDNA clones sequenced to give 3-fold coverage using BigDye Version 3 (Perkin-Elmer, UK) chemistry and an ABI 3700 sequencer.

### Phylogenetic analysis

The identity of the halibut TnT cDNA isolated was assigned by tBLASTx analysis [27] against GenBank and the Medaka (*Oryzia latipes*) EST database [68]. All tetrapod and teleost TnT cDNA sequences were retrieved and their deduced amino acid sequence compared to that of halibut TnTs using ClustalX software [31]. The phylogenetic relationship of halibut TnT genes with other vertebrate *TnT *genes was analysed using the maximum-parsimony method option of PAUP* version 4.0b software [34] with 1000 bootstraps [35] and TnT sequences available from databases (Table 1). *Caenorhabditis elegans *striated muscle TnT (GenBank accession no. NP509076.1) was used as outgroup. All the sequences obtained during this study have been submitted to EMBL/GenBank data library under the accession numbers DQ680172 to DQ680179 (corresponding respectively to sTnThh2, efTnThh, fTnThh-1, fTnThh2, AfTnThh-1, AfTnThh-2, AfTnThh-3 and AfTnThh-4).

**Table 1:** Vertebrate TnT sequences used in phylogenetic analysis

**Specie**	**Gene, abbreviation**	***Database, accession number***
*Homo sapiens*	Slow TnT, sTnT hs	GenBank, AAB3027
	Fast TnT, fTnT hs	GenBank, NP_006748
	Cardiac TnT, cTnT hs	GenBank, NP_000355
*Gallus gallus*	Slow TnT, sTnT ck	GenBank, JC4970
	Fast TnT, fTnT ck	GenBank, AAA49100
	Cardiac TnT, cTnT ck	GenBank, BAA02369
*Coturnix coturnix japonicus*	Fast TnT, fTnT cj	GenBank, P06398
*Xenopus laevis*	Fast TnT, fTnT xl	GenBank, AAM55471
	Cardiac TnT, cTnT xl	GenBank, AAO33406
*Danio rerio*	Intronless TnT, iTnT zf	GenBank, NP_852476
	Slow TnT low MW isoform, sTnTLMW zf	GenBank, BQ259877
	Fast TnT a, fTnTa zf	GenBank, NP_571640
	Fast TnT b isoform 1, fTnTb zf1	GenBank, AF425741
	Fast TnT b isoform 2, fTnTb zf2	GenBank, BC065452
	Cardiac TnT, cTnT zf	GenBank, CAD59126
*Salmo salar*	Fast TnT, fTnT ss	GenBank, AAC24595
*Gadus morhua*	Fast TnT, fTnT gm	GenBank, AAM21701
*Salmo. trutta*	Slow TnT 1S, sTnT 1s st	GenBank, AAB58912
*Fugu rubripes*	Putative slow TnT2, sTnT2 fg	HGMP, M001711
	Intronless TnT, iTnT fg	HGMP, M000253
*Ictalurus punctatus*	Slow TnT 1, sTnT1 ic	GenBank, CK412342
*Orizya latipes*	Slow TnT 2, sTnT2 md	Medaka EST, MF01FSA018J165
	Fast TnT isoform 1, fTnT md1	GenBank, BJ729852
	Fast TnT isoform 2, fTnT md2	GenBank, BJ728074
*Tetraodon nigroviridis*	Putative embryonic fast TnT isoform, efTnT tn	EMBL, CR660426
	Putative larval fast fTnT isoform, fTnT tn2	EMBL, CR658326
	putative adult fast TnT isoform, fTnT tn1	EMBL, CR658422
	Atypical fast TnT isoform 1, AfTnT tn1	EMBL, CR696067
	Atypical fast TnT isoform 2, AfTnT tn2	EMBL, CR675364
	Atypical fast TnT isoform 3, AfTnT tn3	EMBL, CR662746
	Atypical fast TnT isoform 4, AfTnT tn4	EMBL, CR727722
	Atypical fast TnT isoform 5, AfTnT tn5	EMBL, CR673164
*Sparus aurata*	Slow TnT1, sTnT1 sb	GenBank, AY684301
	Slow TnT2, sTnT2 sb	GenBank, AY684302
	Intronless TnT, iTnTsb	GenBank, AY953294
	Embryonic fast TnT isoform, efTnT sb	GeneBank, DQ473445
	Larval fast TnT isoform, LfTnT sb	GeneBank, DQ473444
	Adult fast TnT isoform, afTnT sb	GeneBank, DQ473443
Caenorhabditis elegans	*striated muscle TnT, TnT ce*	GeneBank, NP_509076

### Putative genomic organisation of halibut TnT genes

The putative genomic organisation of isolated halibut *TnT *genes was established *in silico *using the *Tetraodon nigroviridis *genome database [69]. The *Tetraodon *scaffolds giving the most significant hit by tBLASTx analysis [27] with the halibut *TnT *sequences were recovered. Pairwise alignment of halibut and *Tetraodon TnT *cDNA sequences with the selected *Tetraodon *scaffold using Spidey mRNA-to-genome software [32] permitted identification of the putative exon/intron boundaries of the halibut *TnT *genes

### Animal sampling

Atlantic halibut at different developmental stages (Saele et al. 2004) ranging from pre-metamorphic larvae to fully metamorphosed juveniles were obtained from Fiskey (Iceland). Animals (n = 10) were anesthetized in MS-222 (Sigma) and immediately collected for total RNA extraction by preservation in RNAlater (QIAgen, UK) according to the manufacturers instruction. An adult halibut was anesthetised in MS-222 (Sigma-Aldrich, UK) and killed by decapitation and white muscle, red muscle, heart and liver were immediately collected into RNAlater (QIAgen) according to the manufacturers instructions. Alternatively, for *in situ *hybridization and histology anaesthetized halibut larvae and juveniles were fixed in paraformaldehyde (4% PFA) at 4°C overnight. Samples were subsequently washed twice for 5–10 minutes with PBT and stored in 100% methanol at 4°C. Samples where embedded in paraffin and 5 μm post-anal transverse section were made from each animal.

For radioimmunoassay five individual samples were collected for each stage, anesthetised in MS-222 (100ng/mL, Sigma-Aldrich) and immediately frozen in dry ice.

### Total RNA extraction

Total RNA was extracted from whole-body metamorphosing larvae and 100 mg of adult halibut tissue using Tri reagent (Sigma-Aldrich) according to the manufacturer's instructions, quantified in a GeneQuant (Amersham Biosciences) spectrophotometer and stored at -80°C until use.

### Northern Blot

Three micrograms of total RNA obtained from adult halibut white muscle, red muscle, heart and liver where fractioned on a 1.5% agarose/5.5% formaldehyde gel which was run in 1× MOPS. RNA was transferred to nylon Hybond-N membranes (Amersham Biosciences) with 10× SSC overnight and cross-linked using UV light (Stratalinker, Stratagene). Hybridisations were carried out using 3'UTR probe prepared from each cloned halibut TnT gene (~10 μg) by digestion for 2 hours at 37°C with 10U of appropriate restriction enzyme (Promega) and 1× buffer (Table 2). The resulting DNA for probes was purified by electrophoresis followed by extraction of DNA using the GFX gel band extraction kit (Amersham Biosciences).

**Table 2:** Restriction enzymes used to produce 3'UTR probes for northern blot hybridization.

**Gene**	**Restriction enzyme**	**Probe size (bp)**
**fTnThh**	*PstI*/*XhoI*	492
**AfTnThh**	*PstI*	364
**sTnT2hh**	*BtgI/EcoRV*	276

Individual membranes were hybridized overnight at high stringency (65°C in 6×SSC, 5× Denharts solution, 100 μg/mL tRNA and 0.1%SDS) with its respective ^32^P-dCTP-labeled halibut *TnT *probe. The membranes were then washed twice for 30 minutes at room temperature (1×SSC and 0.1%SDS) followed by two 30 minute high stringency washes (65°C in 1×SSC and 0.1%SDS) and exposed at -80°C to Biomax MS film (Kodac, USA).

### Semi-quantitative RT-PCR analysis of TnT expression during halibut metamorphosis

In order to determine expression of halibut *TnT *genes during metamorphosis a semi-quantitative RT-PCR assay was developed. For that, 0.5 μg of total RNA were DNased with the Ambion DNA Free kit (Ca, USA), according to the manufacturers instruction, and used for first strand cDNA synthesis which was carried out in a 20 μL volume using 0.05 M Tris-HCl, pH8.3, 0.075 M KCl, 3 mM MgCl_2_, 0.01 M DTT, 1 mM dNTP, 5 pmol/μl random hexamer primers, 4U of RNAse inhibitor (Promega, UK) and 10U of Superscript II reverse transcriptase (Invitrogen, UK). Synthesis reactions were carried out in an iCycler thermocycler (Perkin Elmer) for 10 minutes at 25°C followed by 50 minutes at 42°C and heating for 2 minutes at 70°C terminated synthesis. Five individual cDNA synthesis reactions corresponding to five individual animals per stage were performed.

Initial RT-PCR amplifications with primers specific for each halibut *TnT *gene were conducted to determine optimal cycle number and ensure that amplification occurred in the logarithmic phase of the reaction. The expression of *18s ribosomal RNA *(*rRNA*) was used as an internal standard for normalisation.

RT-PCR analysis of halibut *TnT *genes was carried out in a 25 μl reaction volume containing ~20 ng of cDNA for each sample and 1.5 mM MgCl_2_, 0.1 mM dNTP's, 1 pmol/μl of halibut specific TnT gene forward and reverse primer (Table 3) and 0.6U *Taq *polymerase (Sigma-Aldrich). Primers for all the halibut TnT genes analysed were selected to amplify the entire N-terminal region, which in terrestrial vertebrates [4] and sea bream [5,6] undergoes alternative splicing. The forward primer was located in the 5'UTR region of the isolated halibut *TnT *cDNAs. The reverse primer was designed in a constitutively expressed region of the halibut *TnT *cDNAs.

**Table 3:** Primer sequence and concentration used for RT-PCR analysis of fTnThh, TnThh and sTnT2hh during halibut metamorphosis.

**Gene**	**Forward Primer**	**Reverse Primer**
**fTnThh**	TCTCAGGTTGCAAAGTCCAC	GACGCTTCTCAATCCTGTCC
**AfTnThh**	CTCTGAGGTGTGAAGTCTG	CTCGACGCTTCTCAATTCGATC
**sTnT2hh**	ATCTTGCTGAGCTCATTCAT	ACGCTGATCCTCCATCTCC

The PCR reactions were performed in an iCycler (Perkin Elmer) thermocycler, using the following cycle; 1 minute at 95°C followed by 27 cycles, for *fTnThh *and *sTnT2hh*, or 28 cycles, for *AfTnThh *of; 30 seconds at 95°C, 1 minute at 56°C and 30 seconds at 72°C, followed by a final step of 1 minute at 72°C. Negative reactions without sample cDNA were also performed.

Amplification of the housekeeping gene *18s rRNA *used for normalisation was carried out as described above using 1 pmol/μl of forward and reverse primer (5'-TCAAGAACGAAAGTCGGAGG-3' and 5'-GGACATCTAAGGGCATCACA-3' respectively). The thermocycle utilised was: 1 minute at 95°C followed by 16 cycles of 30 seconds at 95°C, 1 minute at 56°C and 30 seconds at 72°C, followed by a final step of 1 minute at 72°C. All RT-PCR reaction products were fractionated on 2.5% agarose gels and analysed by densitometry using LabWorks software, version 4.5 (Ultra-Violet Products Cambridge, UK). Results are expressed as the mean and standard error of five independent samples.

### In situ hybridisation

The developmental ontogeny of halibut muscle was characterised using 5 μm post-anal transverse section from several larvae from each stage. Several sections per fish were dewaxed, rehydrated and stained using haematoxylin and eosin and mounted in DPX reagent (Sigma-Aldrich).

The spatial-temporal expression pattern of halibut *fTnT *gene, and its *efTnT *isoform, *sTnT2 *and *AfTnThh *in metamorphosing halibut larvae and post-metamorphosed juveniles was investigated by *in situ *hybridisation using specific digoxygenin riboprobes. For *fTnThh *gene expression a 582 bp riboprobe for the conserved 3'UTR region in all fTnThh isoforms was generated. The expression of *efTnThh *isoform was established by generating a riboprobe complementary to the embryonic/larval exon (aa 12 to 68). *AfTnThh *gene expression was established using a 641 bp riboprobe complementary to the constitutive 3' coding region and the 3'UTR of the *AfTnThh *cDNA. Riboprobes were generated by linearising *AfTnThh *cDNA clones with the appropriate restriction enzyme for 1.5 h at 37°C and isolating the linearised vector by phenol (pH 8) extraction and precipitation in 3 M sodium acetate (pH 5) and ethanol overnight at -20°C.

Specific riboprobes were generated by *in vitro *transcription using the linearized vector as template and was carried out using 20U of T7 RNA polymerase or SP6 RNA polymerase in the case of *efTnThh*, in transcription buffer (Promega) with 1 μl of digoxigenin-RNA labelling mix (Roche Diagnostics, Mannheim, Germany), for 1.5 h at 37 °C. The reaction was stopped with 2 μl of 0.2 M EDTA. The digoxygenin labelled riboprobes were purified by lithium precipitation and ressuspended in 25 μl of water. Riboprobe purity and concentration were determined by fractionation of reaction products on an agarose gel (1.5%). To assess potential cross hybridization between probes and target sequences dot blots were performed. Each digoxygenin labeled riboprobe was hybridized with all the halibut *TnT *target sequence, no cross hybridization reactions were detected and each probe was found to be specific for its target template.

For *in situ *hybridisation experiments adjacent transverse tissue sections of halibut larvae were dewaxed, rehydrated and then prehybridised at 58°C for 2 h in hybridisation solution without probe (50% formamide, 4× SSC, 1 mg ml-1 torula RNA, 0.1 mg ml-1 heparin, 1× Denhardt's, 0.04% CHAPS). Tissues were then hybridised overnight in a humidified box at 58°C in 100 μl per section of hybridisation solution containing approximately 2 ng μl^-1 ^of the riboprobes. Control sections were pretreated with RNase prior to hybridization with riboprobes or the riboprobes were excluded from the hybridizations.

Stringency washes were 3 × 5 min at 58°C with 2× SSC and 5 min at 58°C in 1× SSC. Tissue sections were then washed 2× 5 min with 2× SSC:0.12% CHAPS at RT, followed by a wash for 5 min in 2× SSC:PTW (1:1, v/v) and finally 5 min in PTW. Blocking was performed by incubation in blocking reagent (Boehringer Mannheim, Germany) with 10% heat inactivated sheep serum, detection of hybridised probe was carried out using sheep anti-digoxigenin-alkaline phosphatase (AP) Fab fragments (1/600) (Roche, Lisbon, Portugal). The chromagens for colour detection were NBT (4-nitroblue tetrazolium chloride) and BCIP (5-bromo-4-chloro 3-indolylphosphate) and colour development was carried out over 2 h at 38°C.

Stained sections from *in situ *hybridisation were rinsed in PBS, fixed for 15 min in 4% formaldehyde at room temperature, rinsed in PBS and mounted in glycerol gelatine. Histological and *in situ *sections were analysed using a microscope (Olympus BH2) coupled to a digital camera (Olympus DP11) linked to a computer for digital image analysis.

### Radioimmunoassay for thyroid hormones

Larval extracts were used to assess T4 and T3 content of whole larvae by radioimmunoassay (RIA) using a double-antibody method under equilibrium conditions.

Frozen larvae (n = 8 per stage) were extracted individually in 50 μl methanol, 200 μl chloroform and 100 μl barbital buffer, centrifuged (3,000 rpm for 30 min at 4°C) and the upper phase removed, lyophilized, reconstituted in assay buffer, heat denatured (75°C for 2 hours) and assayed. Standard curves were prepared with T4 or T3 standards (Sigma-Aldrich) dissolved in 0.1N NaOH and diluted to appropriate concentrations in assay buffer. T3 and T4 assays were conducted in barbital buffer (0.07 M, pH 8.6) and Tris buffer (0.1 M, pH 7.4), respectively, and contained 0.1% BSA and 0.1% sodium azide. In both RIA, either 100 μl of standard or larval extract was added. For both hormones, the total assay volume was 300 μl and included 100 μl of ^125^I-T3 (Amersham Biosciences, Buckinghamshire, UK) or ^125^I-T4 and 100 μl T3 antisera (1:15,000, Sigma-Aldrich) or T4 antisera (1:10,000, Sigma-Aldrich). Antisera were added to all tubes apart from those to determine total count (cpm) and non-specific binding. The T3 and the T4 assays were incubated for 16–24 h at 4°C and subsequently, the free hormone was separated from the bound hormone using precipitation with a second antibody [70].

Two-way analysis of variance (ANOVA) was performed in order to establish if significant differences in the concentration of T4 or T3 were detected between different metamorphic stages. If the two-way ANOVA detected significant differences in T4 or T3 between stages the Holm-Sidak (HSM) multiple comparison analysis was performed to determine which stages have different T4 or T3 levels. Both T4 and T3 concentration values were transformed using the logarithmic function before statistical analysis. Significance was considered if p < 0.05. All statistical analysis was performed using SigmaStat version 3 software (SPSS Corp.)

### T4 treatment and halibut TnT expression

Pre-metamorphic halibut larvae (stage 5) were treated for 10 days with T4 (Sigma-Aldrich). The halibut larvae were maintained in 100 L vessels with sea water at 10.5°C. The waterflow was kept at around 0,3 l/min, with the oxygen level at full saturation and disinfected clay dissolved in water was put in the tanks prior to each feeding to shade the environment and ensure maximum feed uptake and the right distribution of light in the tanks.

The hormone (T4) was administered by feeding the larvae T4-enriched artemia and control animals were fed with unenriched artemia. In order to enrich the artemia a solution (1 mg/ml) of sodium-pentahydrate salt of thyroxin (3-(4-(hydroxy-3,5-diiodophenoxy)-3,5-diiodophenyl)L-alanine, Sigma T2501) was prepared in distilled water. Artemia was enriched using 1 ml of T_4 _stock per liter of artemia (each liter containing 300.000 artemia naupli). T4-enriched artemia was fed to the larvae in the afternoon (2/3 of the total feedings) but in the morning the larvae were fed regular artemia (only 1/3 of the total feedings). The experiment lasted for 10 days. Samples of the artemia were taken and RIA for T4 was performed to validate the uptake of T_4 _by the artemia which contained a T4 concentration of 2 μg T4/g artemia.

At the end of the experiment animals were anesthetized with MS-222 (Sigma-Aldrich) and immediately fixed in RNAlater (Sigma-Aldrich) and kept at -20°C until analysis. Four individual treated and control larvae were used for RNA extraction and first strand synthesis reactions which were carried out as described previously. All experiments and animals collection were performed in accordance with EU legislation for Animal Welfare.

RT-PCR for *fTnThh*, *AfTnThh*, *sTnT2hh *and *18s rRNA *was performed with the primers and conditions described previously. Results are presented as the ratio between the gene of interest and 18s. Results are presented as the mean expression of *fTnThh*, *AfTnThh *and *sTnT2hh *of four individual larvae from control and T4 treated groups. One-way ANOVA was performed to determine if statistically significant differences in halibut *TnT *gene expression occurred between control and T4-treated animals. If one-way ANOVA gave significant differences between the control and the T4-treated group in any of the halibut *TnT *genes analysed a multiple comparison test was performed using the Holm-Sidak multiple comparison test (HSD). All statistical analysis was performed using SigmaStat version 3 software (SPSS Corp.) and significance was considered at p < 0.05.

## Abbreviations

TnT- Troponin T

AfTnT- Atypical fast TnT

fTnT- fast muscle TnT

sTnT- Slow muscle TnT

iTnT- Intronless TnT

TH- Thyroid hormone

bp- base pairs

kb- kilobase

aa- amino acids

kDa- kilodaltons

MHC- Myosin heavy chain

MLC- Myosin light chain

Tm- Tropomyosin

UTR- Untranslated region

stg- stage

## Authors' contributions

MAC contributed to the devise of the experiments and acquisition and analysis of molecular data from the experiments, including all bioinformatics analysis, as well as drafting and revision of the manuscripts. NS carried out histological and microscopical data acquisition and drafting of the manuscript. MAN was responsible for the cDNA library construction, isolation and preliminary characterisation of the halibut incomplete TnT clone described in the methods and material section that was subsequently used as a probe in the isolation of further fTnT and sTnT isoforms. LL was also involved in the cDNA library construction, preliminary characterisation of the halibut TnT clone described in the methods and material section that was subsequently used as a probe in the isolation of further fTnT and sTnT isoforms. GES was responsible for the elaboration of the experiments, data analysis and drafting and revision of the manuscript. DMP was responsible for the elaboration of the experiments, data analysis and drafting and revision of the manuscript. All authors read and approved the final manuscript.
